# On a Certain Form of Hæmoptysis, Unassociated with Pulmonary Tuberculosis

**Published:** 1866-06

**Authors:** Richard Payne Cotton

**Affiliations:** M.D., F.R.C.P. Lond., Physician to the Hospital for Consumption and Diseases of the Chest, Brompton


### ON A CERTAIN FORM OF HÆMOPTYSIS, UNASSOCIATED WITH PULMONARY TUBERCULOSIS.

By RICHARD PAYNE COTTON, M.D., F.R.C.P. Lond. Physician to the Hospital for Consumption and Diseases of the Chest, Brompton.

Hæmorrhage from the lungs, to a greater or less degree, is so frequently found in connexion with a tubercular condition of the chest, that its diagnostic value, as a separate symptom, is very apt to be overrated. In the early period of my attendance at the Brompton Hospital for Consumption, I saw so much hæmoptysis amongst the unmistakably phthisical out-patients, that I was sometimes tempted to the general conclusion that hæmoptysis was, practicably, but another name for phthisis. Subsequent and more extensive observation has, however, very much changed my views in this particular; and I am now fully convinced that hæmoptysis is met with in a very considerable number of non-tubercular cases. Simple pulmonary congestion, whether the result of inflammatory action, or arising from mechanical obstruction consequent upon heart-disease; pneumonia, whether acute or chronic; bronchitis; general plethora, —may at any time give rise to hæmorrhage from the lungs. I have seen, indeed, most active bleeding where some of these conditions have been combined—as in congestive bronchitis in persons of plethoric habit. To this list, congested states of the pharynx, tonsils, and gums might fairly be added; but I wish my present observations to apply to hæmoptysis, not in its literal sense, but as it is now usually employed—as hæmorrhage *from the lungs*. In a future communication I propose to enter upon the various forms which hæmoptysis, under these several conditions, is disposed to assume. In the present instance I am anxious only to draw attention to a not unfrequent, but, so far as I know, little recognized form of non-tubercular hæmoptysis, met with chiefly in the female sex, but sometimes also amongst males, generally in the early period of life.

It may simplify my description of this variety of hæmoptysis if I give a brief account of two or three cases in point.

A young lady, aged 18, recently arrived from a residence in one of the West India Islands, was supposed to be phthisical. I was requested to see her, and report upon the nature of her disease, about which several very conflicting opinions had already been given. She was anæmic, nervous, and out of health; had a dry cough, but had not become thinner; her catamenia were regular, but scanty; her appetite was capricious; and she had had frequent hæmoptysis, which there was every reason to believe did not proceed from either the mouth or fauces. The blood upon examination was found to be thin and watery, of a dark color, free from coagulum, unassociated with either bronchial or salivary secretion, and in general appearance much resembling a mixture of *red-currant jelly and water*. I was informed that this was its general character. Sometimes it had been considerable—as much as half a pint in twenty-four hours; at others, it would not exceed a teaspoonful or two during the same period; sometimes it would be scarcely enough to tinge a pocket-handkerchief, and often it would disappear for days together. This state of things had existed for nearly two years, causing great anxiety to the patient and her friends, from a belief that it was indicative of pulmonary disease. Careful examination of the chest, however, failed to elicit any evidence that such was the case. Rest, change of air, and the tincture of sesquichloride of iron, entirely restored this patient to health. It is now more than three years since I was consulted, and I heard a short time back that the young lady was in perfect health.

A case very similar to this came under my notice two years ago, in consultation with Mr. Humpage, of Upper Seymour-street. A young lady aged 24, had long been delicate, and was supposed by her family to be consumptive. She had become thinner; had had a dry cough for some length of time; and had spat blood. Upon examination, this was found to present an appearance very similar to that described in the preceding case; it looked in fact, more like watery red-currant jelly than anything else. As in the other patient, there were no decided physical signs of tubercular disease; and we came to the conclusion that the patient was not phthisical. Time has justified our diagnosis; Mr. Humpage having lately informed me that the young lady had, for many months, lost the symptom which had caused so much alarm, and was in good health.

Another case, which I shall even more briefly relate, came under my notice about 18 months back. It was that of a young lady, 12 years of age, of slender and somewhat delicate appearance, but free from every symptom of tubercular affection. Mr. J. N. Winter, of Montpeiler-road, Brighton, frequently saw this patient, and quite agreed with me as to the nature of her disease. She first alarmed her parents by spitting up, just after going to bed one night, a considerable quantity of blood. This was found upon examination to be watery and dark-colored—in fact, of the *thin red-currant jelly character* already described. This symptom has recurred at various intervals with more or less intensity, and the child still remains delicate, but without any indications of tubercular disease. At the time of her attacks her tonsils and pharynx are somewhat conjested, her gums spungy, and her fingers even have sometimes exuded a little watery blood, just as one sometimes sees in extreme cases of purpura. It is evident that, in this instance, the blood escapes not only from the mucous membrane of the respiratory passages, but also from that of the throat, tonsils, and gums.

Other cases of this form of hæmoptysis have fallen under my observation; but I shall not specially refer to them in consequence of not knowing their sequel. At least 12 or 13 have happened in my own wards at the Hospital for Consumption. Two only of these were males; the rest were females, generally of delicate and nervous appearance, and under the age of 30. Several had very suspicious symptoms of phthisis; but the physical signs failed to exhibit any evidence of pulmonary tuberculosis, and most of them improved in health under appropriate treatment. In every case the expectoration was of the same general character; sometimes it was mixed more or less with bronchial mucus, slightly tinged perhaps with blood, and sometimes with salivary secretion; but more frequently it was simply watery blood, resembling, as I have described, a mixture of red-currant jelly with water.

The following are the conclusions at which I have arrived from a consideration of the preceding notes:—

1st. There is a form of true hæmoptysis in which the expectoration is of a dark color, and of a more or less watery consistence, bearing a close resemblance to a mixture of red-currant jelly and water.

2d. That such hæmoptysis is of non-tubercular origin, and may proceed from any part of the gastro-pulmonary mucous membrane.

3d. That it is attributable to a morbid and fluid condition of the blood, allied, at least in appearance, to that which is met with in purpura and scurvy.—*London Lancet.*

